# Treatment of Inverted Toe Nail

**Published:** 1835

**Authors:** 


VI. *Treatment of Inverted toe Nail.*—In the Clinical paper from which we have made the foregoing extract we find the following practical directions for the cure of inverted toe nail.

"In the treatment of this affection, let the surgeon first carefully consider the cause, and endeavor to effect its removal. This I have accomplished by interposing between the toes, at their roots, a cylinder of soft rolled linen—perhaps a third of an inch in diameter, or as large as may be necessary to keep the extremities of the toes from pressing upon each other. A shoe is to be worn which shall give abundant room for the toes to expand, but furnish snug support to the instep; for a shoe which is altogether loose upon the foot, will allow the toes to be pushed forward upon the toe of the shoe. The patient must also wear stockings which shall not in the slightest degree compress the toes. If the disease be not far advanced, the parts being relieved of the cause, will soon spontaneously assume their healthy condition. But if there be morbid granulations rising, in which the border of the nail is buried, and this last appears to be creating great irritation, it is necessary to gently raise the border of the nail by seizing the corresponding anterior angle with forceps, and then, with delicate scissors, the free lateral margin which will be found macerated, white, soft, and brittle, should be cut away. To the granulations the nitrate of silver may be applied. The toe-nail should now be scraped thin upon its dorsum, and the patient should be directed every day to lift the border of the nail. I have found no advantage in thrusting anything under the nail to keep it raised, but have seen such substance cause much irritation." p. 390–1.

